# Supplementary material for: Risk factors for delay in age-appropriate vaccinations among Gambian children
Source: BMC Health Serv Res. 2015 Aug 28;15:346. doi: 10.1186/s12913-015-1015-9 (PMC4551385; doi:10.1186/s12913-015-1015-9)
Supplement: Additional file 1: Table S1. — Risk factors for delay in receipt of BCG and DPT3 vaccine. Table S2. Risk factors for delay in receipt of measles and delay of any vaccines. (DOCX 24 kb) [file 12913_2015_1015_MOESM1_ESM.docx]

**Risk factors for delay in age-appropriate vaccinations among Gambian children**

Aderonke Odutola^1^, Muhammed O. Afolabi^1^, Ezra O. Ogundare^1^, Yamu Ndow Lowe-Jallow^2^ Archibald Worwui^1^, Joseph Okebe^1^, Martin O. Ota^1, 3^

Authors Affiliation

^1^Medical Research Council Unit, Fajara, The Gambia

^2^Ministry of Health and Social Welfare, The Gambia

^3^World Health Organization Regional Office for Africa, Brazzaville, Congo

[aaodutola@mrc.gm](mailto:aaodutola@mrc.gm)

[mafolabi@mrc.gm](mailto:mafolabi@mrc.gm)

[tundeyogundare@yahoo.com](mailto:tundeyogundare@yahoo.com)

[ylowe_jallow@hotmail.com](mailto:ylowe_jallow@hotmail.com)

[aworwui@mrc.gm](mailto:aworwui@mrc.gm)

[jokebe@mrc.gm](mailto:jokebe@mrc.gm)

[otama@who.int](mailto:otama@who.int)

Corresponding author: Dr Aderonke Odutola, Medical Research Council Unit, The Gambia, PO Box 273, Banjul, The Gambia. Tel: +220 4495442, e-mail: [aaodutola@mrc.gm](mailto:aaodutola@mrc.gm)

Word count: abstract – 254

Word count text – 2999

# Number of tables – 3

Figure - 1

# ABSTRACT

# Background

# Vaccination has been shown to reduce mortality and morbidity due to vaccine-preventable diseases. However, these diseases are still responsible for majority of childhood deaths worldwide especially in the developing countries. This may be due to low vaccine coverage or delay in receipt of age-appropriate vaccines. We studied the timeliness of routine vaccinations among children aged 12-59 months attending infant welfare clinics in semi-urban areas of The Gambia, a country with high vaccine coverage.

**Methods**

A cross-sectional survey was conducted in four health centres in the Western Region of the Gambia. Vaccination dates were obtained from health cards and timeliness assessed based on the recommended age ranges for BCG (birth–8weeks), Diphtheria-Pertussis–Tetanus (6weeks–4months; 10weeks–5months; 14weeks–6months) and measles vaccines (38weeks–12months). Risk factors for delay in age-appropriate vaccinations were determined using logistic regression. Analysis was limited to BCG, third dose of Diphtheria-Pertussis -Tetanus (DPT3) and measles vaccines.

**Results**

Vaccination records of 1154 children were studied. Overall, 63.3% (95%CI 60.6–66.1%) of the children had a delay in the recommended time to receiving at least one of the studied vaccines. The proportion of children with delayed vaccinations increased from BCG [5.8% (95%CI 4.5–7.0%)] to DPT3 [60.4% (95%CI 57.9%-63.0%)] but was comparatively low for the measles vaccine [10.8% (95%CI 9.1%-12.5%)]. Mothers of affected children gave reasons for the delay, and their profile correlated with type of occupation, place of birth and mode of transportation to the health facilities.

**Conclusion**

Despite high vaccination coverage reported in The Gambia, a significant proportion of the children’s vaccines were delayed for reasons related to health services as well as profile of mothers. These findings are likely to obtain in several countries and should be addressed by programme managers in order to improve and optimize the impact of the immunization coverage rates.

**Key words:** Timeliness, vaccination, protection, vaccine preventable diseases, children.

**Abbreviations:** BCG, Bacille-Calmette-Guerin; DPT, diphtheria-pertussis-tetanus; EPI, Expanded Programme on Immunization; Hib, *Haemophilus Influenzae* type b; IWC, infant welfare clinics; OPV, oral polio vaccine; PCV, pneumococcal conjugate vaccine; UNICEF, United Nations International Children's Emergency Fund; WHO, World Health Organisation.

# BACKGROUND

Immunization is one of the most effective public health interventions against vaccine-preventable diseases. The vaccination schedule varies in different parts of the world and is determined by a combination of the epidemiology of the targeted infections and the ability of these vaccines to induce the required immune response in the child. Thus the vaccine schedules are designed to protect the children when they are most vulnerable to the targeted infections. Consequently, the World Health Organisation (WHO) provides guidelines on the age at which each vaccine should be given and the intervals between vaccinations. These recommended vaccination schedules reduce the risk of the individual child contracting the disease under consideration whilst contributing to achieving the general herd immunity that protects against outbreaks of the disease in the population[[1](#_ENREF_1)]. Therefore poor or non-adherence to these schedules could potentially reverse the benefits of immunizations at individual and community levels[[2](#_ENREF_2), [3](#_ENREF_3)], and underlines the importance to adhere to the age-appropriate schedules for vaccinations. In fact, one of the recommendations of WHO/UNICEF Global Immunization and Vaccine Strategies is to improve surveillance on deviation from age-appropriate immunizations in low- and middle-income countries like Gambia[[1](#_ENREF_1), [4](#_ENREF_4)].

Evidence has shown that these vaccines have reduced the morbidity and mortality associated with childhood infectious diseases[[5](#_ENREF_5), [6](#_ENREF_6)]. However, vaccine-preventable diseases are still responsible for over three million childhood deaths each year globally especially in low income countries[[7](#_ENREF_7), [8](#_ENREF_8)]. Some of the major factors that determine this trend include: low proportion of population immunized which is responsible for herd immunity, challenges with cold chain logistics and gaps in the timing of the vaccine administration which create a period of inadequate protection to the child. When a large number of children have gaps in the timing of vaccination or not vaccinated at all, the result is a significant population susceptible to disease as well as capable of propagating transmission of the disease. The proportion of children that received a particular dose of a vaccine is used to determine the immunization coverage rate. WHO has suggested including age-appropriate vaccination as another indicator of evaluating the quality of immunization services[[1](#_ENREF_1), [9](#_ENREF_9)]. Researchers assessing timeliness of vaccination in some low and middle-income countries reported substantial delay in the receipt of age-appropriate vaccinations[[4](#_ENREF_4), [10](#_ENREF_10)].

In 1979, The Gambia introduced the Expanded Programme on Immunization (EPI) comprising vaccines against tuberculosis, diphtheria, pertussis, tetanus, measles and yellow fever. Hepatitis B and *Haemophilus influenzae* type b (Hib) vaccines were introduced in 1986 and 1997 respectively, and recently the rotavirus and pneumococcal conjugate (PCV7 in 2009 and PCV13 in 2011) vaccines have been added (Table 1). Recent WHO/UNICEF coverage estimates for most vaccines are over 80% - BCG (88%), DPT3 (83%), OPV3 (84%) and Measles (84%)[[3](#_ENREF_3), [11](#_ENREF_11)].The Gambia EPI is one of the most successful in sub-Saharan Africa with up to ten vaccines being administered with high coverage rates. Vaccination coverage in The Gambia is also very high for most of these: 95% for BCG, 90% each for third dose of DPT, hepatitis B and Hib vaccines while 85% coverage was reported for measles vaccine[[12](#_ENREF_12)]. Despite these impressive immunization coverage rates vaccine preventable diseases still occur, which might be related to timeliness[[13](#_ENREF_13), [14](#_ENREF_14)]. For instance, in one study where infants were expected to receive their routine EPI vaccines at ages 2, 3 and 4 months, the average time of receipt of two doses of the vaccine was 4.75 months[[15](#_ENREF_15)]. Other studies have reported that there is an increased delay in the time a vaccine is given compared to when it is due as children get older, with the longest delay being between DPT3 and measles[[16](#_ENREF_16), [17](#_ENREF_17)].

Although a number of factors responsible for low vaccination coverage in Africa [[5](#_ENREF_5), [18](#_ENREF_18), [19](#_ENREF_19)] have been identified, only very few studies have examined the risk factors for delay in age-appropriate vaccination[[13](#_ENREF_13), [18](#_ENREF_18), [20](#_ENREF_20)]. Recently, two studies from the Gambia have reported on childhood vaccination; one on the predictors of vaccination in rural Gambia [[21](#_ENREF_21)] and the other looked at the coverage and timeliness of childhood vaccination [[22](#_ENREF_22)]. Given the role age-appropriate vaccination and coverage have on the vaccine preventable diseases, this study assessed the timeliness of vaccination for BCG, OPV1-3, DPT1-3 and measles vaccines, risk factors and reasons for delayed childhood vaccinations during the first 12 months of life.

# METHODS

**Study setting**

This study was conducted from January to June 2011 at the infant welfare clinics (IWC) of Fajikunda, Serrekunda and Sukuta Health Centres and Jammeh Foundation for Peace Hospital in the Western Region of The Gambia. These facilities serve an area of about 1,705 square Km with a population of about 392,000 people of which the majority are farmers or civil servantsThe IWC services include immunization services, growth monitoring, general health and nutrition education. In The Gambia, every newborn is given a health card where EPI vaccinations and dates of administration of the vaccines are recorded by immunization officers. The health cards also contain information such as birth record, vaccination schedules and monthly weight measurements for growth monitoring. The mothers are allowed to take the health card home and present it at all clinic visits.

**Study Design and Data Collection**

This was a cross-sectional survey targeting children aged between 12 and 59 months attending the health centres with their health cards on the survey day. The survey team was made of two clinicians and four field assistants who had experience in epidemiological surveys and were familiar with immunization dynamics in the study areas. The field assistants gave sensitisation talks about the study to the mothers attending the immunization clinics with their children. After this, the field assistants identified potentially eligible mother-child pairs and further individualised consent discussions were held. Consequent upon granting a written informed consent, the clinicians and field assistants obtained the following information from the child’s health card: date of birth (DOB), birth order, sex, place of birth and dates of the administered vaccines. This was followed by administration of a purpose-designed, structured questionnaire to the mothers. The questionnaire covered information on mother’s age, residence, parent’s level of education, parent’s concerns and perception about the vaccine benefits. In addition, mothers of children with delayed vaccination schedules were probed to give reasons for the delays. As the sample size was not stratified by study sites and age-groups of the target population, consenting mothers were enrolled in each recruitment site irrespective of the child’s age while children without verifiable records were excluded from this study.

# Sample size calculation

# Based on the proportion of children who had delayed vaccinations in Rietvlei, South Africa (42%)[[13](#_ENREF_13)], a precision of 3% and a 95% confidence interval, a sample size of 1040 children was required. After adjusting for attrition rate of 10% the sample size was approximately 1144.

# Definition of terms

# A complete vaccination schedule was defined as having received a dose of BCG (birth – 8 weeks), three doses of DPT-Hib-HBV [DPT1/OPV1 (6 weeks – 14 weeks); DPT2/OPV2 (10 weeks – 18 weeks); DPT3/OPV3 (14 weeks – 24 weeks)] and a dose of measles vaccine (38 weeks – 52 weeks) respectively (Table 1). The age at vaccination was recorded in days (date of vaccination minus date of birth). Timeliness of vaccination of a particular antigen was assessed against the WHO recommended range as already indicated above. Timeliness was categorised as follows: (a) too early (vaccine was received earlier than the recommended age); (b) timely (vaccine was received within the recommended period above); (c) delayed if received after the window period.

# Data Analysis

Data were double entered into a Microsoft Access database and analysed using Stata 12.0 (College Station, Texas 77845 USA). Categorical variables were presented using proportions and continuous variables described using an appropriate measure of dispersion: means (standard deviations) or medians (Inter Quartile Range). Logistic regression was used to analyze factors associated with delay in receipt of each vaccine and delay. We did not include maternal age in multivariate analysis because it was correlated with birth order of the child (r=0.66, p<0.001).

# Ethical considerations

The study was approved by the Gambian Government/Medical Research Council Joint Ethics Committee. A written informed consent was obtained from the respondent before the questionnaires were administered.

# RESULTS

# Socio-demographic characteristics of children and their parents

A total of 1477 mothers were approached to join the study, 1448 gave consent giving a response rate of 98.0%. These mothers were interviewed but forty-two were not included in the analysis due to the following reasons: no vaccination records (15), vaccination cards were defaced and dates were illegible (27). A total of 252 were dropped from analysis as they were younger than 12 months of age. Of the 1154 children, 258 were from Fajikunda Health Centre, 483 from Jammeh Foundation for Peace Hospital, 194 from Sukuta Health Centre and 219 from Serrekunda Health Centre. The median (IQR) age of the children analysed was 19 (15, 30) months, and 601 (52.1%) were boys (Table 2a). The mean age of the mothers was 27.0±5.7 years and 67 % of the mothers had at least primary school education (Table 2b). Most of the respondents (1038/1154; 90.0%) knew that the vaccines can protect a child from contracting infections. When asked about concerns with vaccinations, 20.0% (230/1149) reported fever following vaccinations and 31.2% (358/1399) of the respondents reported pain at injection site. Ninety-four children had missing dates on their health card for at least one of their vaccines.

# Proportions of children who had age-appropriate vaccinations

The proportion of children who had timely vaccinations varied for the different vaccines (Figure 1). Those who were timely vaccinated were: BCG 94.3% (95% CI 93.0 – 95.6%); DPT1 78.4% (95%CI 76.0 – 80.8%), DPT2 49.7% (95% CI 46.8 – 52.6%), DPT3 39.6% (95% CI 36.8 – 42.4%), OPV1 74.6% (95% CI 72.0 – 77.1%), OPV2 50.0% (95% CI 47.1 – 52.9%), OPV3 40.6% (95%CI 37.7 – 43.4%), for measles 80.8% (95% CI: 78.5 – 83.1%). One hundred and eighty nine (13.4%) children had their vaccinations before the scheduled time: 47 (4.1%) for DPT1, 20 (1.7%) for DPT2, 6 (0.5%) for DPT3 and 84 (7.4%) for measles). Overall, 63.3% (95% CI 60.6 – 66.1%) of the subjects had delayed vaccination of at least one of their vaccines. Two-thirds of the children received BCG before 2 weeks of age. The median (IQR) age at vaccination is shown in table 3.

# Risks factors for delay in age-appropriate vaccinations

The reasons given by caregivers for delayed vaccination were long waiting times at the health facilities (22.5%), lack of (22.5%) or forgotten (17.5%) vaccination appointments, ill-health of either mother or child on the appointment day (5.0%). None of the respondents reported parental or family objections to vaccination as a reason for delay. Majority of the responders liked the monthly vaccination schedule because it was easy to remember. About 60% and 62% of the mothers of children who had experienced delay in the receipt of any vaccine had concerns about fever and pain at the injection site respectively post-vaccination.

Furthermore, the characteristics of caregivers with delayed child vaccination in univariate analysis revealed that unemployed and illiterate mother was significantly associated with delay in receiving BCG and, only unemployment status remained significant in multivariate analysis (see supplemental table). Children of mothers who were civil servants were less likely to receive their BCG vaccines later compared to children of mothers who were unemployed (OR 0.17 95%CI 0.02- 1.22) (Table 4).

Mother’s occupation, father’s education, place of birth, mode of transportation, birth order, delays in receipt of DPT1 and DPT2 were strongly associated with delays in receiving DPT3 in a univariate analysis (see supplemental table) but in the multivariate analysis, mothers who were traders, children born at home, those who went to the clinic by public transport and delay in receipt of DPT2 were independently associated with delayed DPT3 (Table 4).

Age group, father’s education, family size, place of birth, mode of transportation to the clinic, number of stops (number of times parents/caregivers had to change public transport vehicles before they got to the health centres), delays in receipt of DPT1, DPT2 and DPT3 were associated with delay in receipt of measles vaccine in the univariate analysis (see supplemental table) while in the multivariate analysis illiterate fathers, those who went to the clinic by private transport delays in receipt of DPT1 and DPT3 were independently associated with delay in receipt of the vaccine (Table 4). Children of illiterate fathers were twice more likely to have had delay in receipt of measles vaccine compared to children whose fathers had at least primary school education (OR 0.54; 95% CI 0.35 – 0.82).

In multivariate analysis, factors associated with delay in receipt of any of the routine vaccines included decreasing birth order, children born at home and taking public transport to the clinic were independently associated with delay in receipt of any of the routine vaccines (Table 4). Children with increasing birth order had 30% the odds of delay in receipt of any of the vaccines compared to children birth order less than 2 (OR 1.27; 95%CI 0.99 – 1.64). Children who were born at home were more likely to have a delay in receipt of any of the vaccines compared to those who were born in a health facility (OR 1.66; 95%CI 1.16 – 2.37). Children whose caregiver came to the clinic in public transport were more likely to be delayed in receiving any of the vaccines (OR 1.45; 95%CI 1.12 – 1.86).

# DISCUSSION

# This study identified that about two-thirds of children aged between 12 and 59 months attending the health centres in the Western Region of The Gambia for immunization had experienced delay in the receipt of at least one of their vaccines. The proportion of children who had delayed vaccination was least for BCG and highest for DPT3 and OPV3. The reasons given by the caregivers for their children having delayed vaccination included lack of appointment date, long waiting times at the health facilities, forgotten appointment date and ill-health of either mother or child. The independent risk factors associated with delay in receipt of any vaccines were increasing birth order, being born at home and taking public transport to the clinic. Factor associated with delay in receipt of BCG was mothers’ occupation and those for DPT were mothers’ occupation, being born at home and taking public transport to the clinic while for measles children with illiterate fathers.

# Delay in the receipt of all age-appropriate vaccines was seen in this study and all doses were affected. A similar finding was observed in one study where infants were expected to receive their routine EPI vaccines at ages 2, 3 and 4 months, the average time of receipt of two doses of the vaccine was 4.75 months[[15](#_ENREF_15)]. In our study, the delay in receipt of BCG was minimal which may be due to the fact that BCG vaccines are available at the health centres where most of the deliveries took place. There was a steady decline in the receipt of age-appropriate vaccines from BCG to DPT3/OPV3. Other studies have reported an increased delay in the time a vaccine is given compared to when it is due as children get older, with the longest delay being between DPT3 and measles[[16](#_ENREF_16), [17](#_ENREF_17)]. This may be explained by the fact that the older the child gets, the more pre-occupied the mother or caregiver gets with other domestic/family activities thereby not remembering vaccination appointments for the child. Also as shown in this study, if the child had some side effects like fever, pain or swelling at the injection site following previous vaccinations, the mother may not be inclined to go for subsequent doses. Of note is the fact that the third dose of OPV was significantly delayed in about two-thirds of the participants, given that the routine immunization would be required to sustain the impact of the oral polio supplemental immunization. Our data are similar to the findings of other studies[[16](#_ENREF_16), [20](#_ENREF_20), [24](#_ENREF_24)].

# Only about one-third of the children surveyed had received all their vaccines on-time. This is similar to the findings of other researchers[[4](#_ENREF_4), [14](#_ENREF_14), [25](#_ENREF_25)]. This means a large proportion of children are unprotected or inadequately protected against these vaccine-preventable diseases for variable lengths of time. Despite the high vaccine coverage, this may be one of the reasons why under-five mortality is still quite high in The Gambia[[26](#_ENREF_26)]. The infant and under-five mortality rates in The Gambia are 57/1000 and 103/1000 live births respectively[[23](#_ENREF_23)]. In a study assessing possible reductions in childhood mortality in The Gambia[[26](#_ENREF_26)], pneumonia accounted for 11% of deaths among infants aged 0–11 months and for 9% among children aged 1–4 years; no measles death was reported. About 80% of the children received measles vaccine in a timely fashion. This may be due to the fact that mothers tend to remember this as it is the last scheduled vaccination visit for the primary vaccines. This finding is contrary to what was reported in other studies[[16](#_ENREF_16), [24](#_ENREF_24)]. The reason for this difference is not immediately known. It may be due to mothers’ knowledge of the severity of measles from past outbreaks in the country[[27](#_ENREF_27)].

Interestingly, majority (~60%) of the reasons for delayed vaccination by the caregivers blamed the health centre staff for contributing to it by having not given appointment for the next vaccination date or discouraged by the long period of time spent to get their children vaccinated in their previous visits to the clinics. These reasons appear plausible and are supported by the fact that the delay increases from DPT1/OPV1 to DPT3/OPV3, implying that EPI workers are not emphasizing subsequent clinic visits enough to mothers and/or the mothers have had increasing bad experiences from the facilities and health workers in previous visits. Further support for these reasons come from the profile of the caregivers with delayed child vaccination, including low literacy, far distance of clinic from home, delivery at home and use of public and commercial transportation to the clinics. This profile represents a group of women with poor understanding of the importance of vaccination, low socio-economic status who can easily be discouraged to vaccinate their children by the cost and attitude of health care workers.

Unemployed mothers were likely to have their children’s early vaccinations delayed. Unemployment in the context of the setting of our study is likely to correlate with low education, and possibly inability to appreciate and demand vaccination for child[[13](#_ENREF_13), [20](#_ENREF_20)]. It may also be that these unemployed women were fully engaged at home with domestic and farming chores hence they tended to forget their children’s vaccination appointments. Also, unemployed mothers may not be financially empowered to come to the health facilities in good time. It is not surprising that children who were born at home were likely to have delay in the receipt of age-appropriate vaccines. This may be due to poor health seeking behaviour of the mother and social determinants which limit the decision-making of pregnant women in patriarchal societies as reported in other settings[[28](#_ENREF_28), [29](#_ENREF_29)]. This is similar to what was found in South Africa[[10](#_ENREF_10)]. Mode of transportation was an independent risk factor for delayed vaccination. Children of mothers who had private transport were one third less likely to have delay vaccination compared with children whose mothers had to trek to the health facilities; whereas children whose mothers had to take public transport to the health facilities were more likely to have delayed vaccination. By implication most of the mothers who trekked to the health centre, most likely lived close to the health centre and did not require to take public transport. It is not surprising to see that delay in receipt of previous vaccines was associated with delay in receipt of the next dose.

Our data and others [[4](#_ENREF_4), [13](#_ENREF_13), [14](#_ENREF_14), [16](#_ENREF_16), [20](#_ENREF_20), [24](#_ENREF_24), [30](#_ENREF_30)] show that having a high proportion of children with delayed vaccination is likely to be the case in most countries. Appropriate measures for each setting have to be taken to mitigate against the factors responsible for this weakness in the vaccine delivery system. Mobile telephony has been used to deliver SMS to improve vaccination coverage[[31](#_ENREF_31)]. This method could be adopted to remind mothers of the vaccination dates. The health workers should be trained on the need to have positive attitudes to mothers, developing the most effective quick service delivery at vaccination stations and giving appointments of next clinic visits in the language mothers will understand. The need for having health facilities within reach of the community, or outreach visiting stations where vaccinators visit at scheduled days of the week cannot be overemphasized. This is already being done in Gambia. After the completion of the study, we educated the mothers about the importance of bringing their children for vaccinations as scheduled. Practical ways of addressing the barriers militating against timely vaccination were also discussed with the mothers. The EPI officers were encouraged to always remind the mothers of the subsequent vaccination dates.

A limitation of our study is that it is a health facility-based survey instead of community-based that would have been more representative of the children population. However, the community-based data are likely to be worse given that the parents of the infants seen at the health facilities are likely to have good health seeking behaviour and confident to be reasonably compliant with the vaccination schedules to appear at the health facility. Thus, our findings are still significant and relevant in drawing attention to this often neglected aspect of timeliness of vaccination. We did not assess other childhood vaccines like Hib and HBV vaccines separately because they are given at the same time as DPT as a single injection, so their results are likely to be similar to DPT vaccines. Also children with unverifiable records were excluded from analysis therefore we might have missed some children with delay vaccinations. Even though vaccines are given in the second year of life in The Gambia, we did not study the timeliness of these vaccines.

Another weakness of this study is skewness of the age-groups of study infants. In our setting, after the first two years of life the number of children attending the IWC decreases drastically. A community-based study would have almost all age groups represented equally.

# Conclusion

An unacceptably high proportion of the infants seen in these health centres in Gambia experience delay in the receipt of at least one of their vaccines later than recommended. This weakness in vaccination service delivery is common to many countries and appropriate steps must be taken urgently in order to optimize the impact of increasing vaccination coverage.

# Acknowledgements

We thank the Gambian government, the EPI programme of The Gambia, and staff of Fajikunda, Serrekunda and Sukuta Health Centres for their collaboration. We appreciate the Fajikunda and Sukuta field teams for their excellent field work. We thank Isatou Ndow, Amie Janha and Nene Cham for data entry assistance. We are particularly grateful to the parents and guardians that allowed their infants to participate in this study.

# Competing Interests

# The authors declare no competing interestsAuthors Contributions

AO conceived the study, participated in the design, was involved in collection, analysis and interpretation of data and drafted the manuscript. MOA participated in the design, was involved in data collection and revision of manuscript. EOO participated in the design, was involved in data collection and revision of manuscript. YNL participated in the design and revision of manuscript. AW was involved in collection and management of data and revision of manuscript. JO conceived the study, participated in the design and revision of manuscript. MOO conceived the study, participated in the design and revision of manuscript. All authors read and approved the final manuscript.

# Figure legends

Table 1. The Gambian Expanded Programme on Immunization schedule (2011).

Table 2a. Socio-demographic characteristics of participants (b) their parents in The Gambia.

Table 3: Median (IQR) age at vaccination for each vaccine in The Gambia.

Table 4. Risk factors for delay in receipt of BCG, DPT3, Measles and delay of any vaccines in The Gambia.

Figure 1. Proportions (95% CI) of children who received each vaccine early, on time and late

# REFERENCES

1. **WHO recommendations for routine immunization** [<http://www.who.int/immunization/policy/immunization_schedules/en/>]

2. Heiniger U Z: **Immunisation rates and timely administration in pre-school and school-aged children**. *European Journal of Pediatrics* 2006, **165**:124-129.

3. Clark A, Sanderson C: **Timing of children's vaccinations in 45 low-income and middle-income countries: an analysis of survey data**. *Lancet* 2009, **373**(9674):1543-1549.

4. Suarez-Castaneda E, Pezzoli L, Elas M, Baltrons R, Crespin-Elias EO, Pleitez OA, de Campos MI, Danovaro-Holliday MC: **Routine childhood vaccination programme coverage, El Salvador, 2011-In search of timeliness**. *Vaccine* 2014, **32**(4):437-444.

5. Odusanya OO, Alufohai EF, Meurice FP, Ahonkhai VI: **Determinants of vaccination coverage in rural Nigeria**. *BMC Public Health* 2008, **8**:381.

6. WHO U, World Bank: **State of the world’s vaccines and immunization.** In: *Geneva: World Health Organization.* 2009.

7. UNICEF: *The State of the WOrld's Children, 1993* 1993.

8. Levine OS, Bloom DE, Cherian T, de Quadros C, Sow S, Wecker J, Duclos P, Greenwood B: **The future of immunisation policy, implementation, and financing**. *Lancet* 2011, **378**(9789):439-448.

9. **WHO recommendations for routine immunization** [<http://www.who.int/immunization/policy/Immunization_routine_table2.pdf?ua=1>]

10. Akmatov MK, Mikolajczyk RT: **Timeliness of childhood vaccinations in 31 low and middle-income countries**. *Journal of epidemiology and community health* 2012, **66**(7):e14.

11. **Global routine vaccination coverage,2011**. *Releve epidemiologique hebdomadaire / Section d'hygiene du Secretariat de la Societe des Nations = Weekly epidemiological record / Health Section of the Secretariat of the League of Nations* 2012, **87**(44):432-435.

12. (WHO) TUNCsFUWHO: **Diarrhoea : Why children are still dying and what can be done**. In*.* Switzerland: World Health Organization; 2009.

13. Fadnes LT, Nankabirwa V, Sommerfelt H, Tylleskar T, Tumwine JK, Engebretsen IM: **Is vaccination coverage a good indicator of age-appropriate vaccination? A prospective study from Uganda**. *Vaccine* 2011, **29**(19):3564-3570.

14. Luman ET, Barker LE, Shaw KM, McCauley MM, Buehler JW, Pickering LK: **Timeliness of childhood vaccinations in the United States: days undervaccinated and number of vaccines delayed**. *JAMA* 2005, **293**(10):1204-1211.

15. Cutts FT, Zaman SM, Enwere G, Jaffar S, Levine OS, Okoko JB, Oluwalana C, Vaughan A, Obaro SK, Leach A *et al*: **Efficacy of nine-valent pneumococcal conjugate vaccine against pneumonia and invasive pneumococcal disease in The Gambia: randomised, double-blind, placebo-controlled trial**. *Lancet* 2005, **365**(9465):1139-1146.

16. Sadoh AE, Eregie CO: **Timeliness and completion rate of immunization among Nigerian children attending a clinic-based immunization service**. *J Health Popul Nutr* 2009, **27**(3):391-395.

17. Onyiriuka A: **Vaccination default rates among children attending a static immunisation clinic in Benini, city, Nigeria**. *J Bio Med Res* 2005, **4**:71-77.

18. Cutts FT, Rodrigues LC, Colombo S, Bennett S: **Evaluation of factors influencing vaccine uptake in Mozambique**. *Int J Epidemiol* 1989, **18**(2):427-433.

19. Ettarh RR, Mutua MK, Kyobutungi C: **Ethnicity and delay in measles vaccination in a nairobi slum**. *Trop Med Health* 2012, **40**(2):59-62.

20. Le Polain de Waroux O, Schellenberg JR, Manzi F, Mrisho M, Shirima K, Mshinda H, Alonso P, Tanner M, Schellenberg DM: **Timeliness and completeness of vaccination and risk factors for low and late vaccine uptake in young children living in rural southern Tanzania**. *Int Health* 2013, **5**(2):139-147.

21. Payne S, Townend J, Jasseh M, Lowe Jallow Y, Kampmann B: **Achieving comprehensive childhood immunization: an analysis of obstacles and opportunities in The Gambia**. *Health policy and planning* 2014, **29**(2):193-203.

22. Scott S, Odutola A, Mackenzie G, Fulford T, Afolabi MO, Lowe Jallow Y, Jasseh M, Jeffries D, Dondeh BL, Howie SR *et al*: **Coverage and timing of children's vaccination: an evaluation of the expanded programme on immunisation in The Gambia**. *PLoS One* 2014, **9**(9):e107280.

23. Department of Central Statictics TG: **2003 Population Census**. In*.*; 2005.

24. Babirye JN, Engebretsen IM, Makumbi F, Fadnes LT, Wamani H, Tylleskar T, Nuwaha F: **Timeliness of childhood vaccinations in Kampala Uganda: a community-based cross-sectional study**. *PLoS One* 2012, **7**(4):e35432.

25. Odusanya O: **Age-appropriate immunization coverage in a rural community in Edo state, Nigeria.** *J Nig Inf Cont Assn* 2000, **3**:9.

26. Jasseh M, Webb EL, Jaffar S, Howie S, Townend J, Smith PG, Greenwood BM, Corrah T: **Reaching millennium development goal 4 - the Gambia**. *Tropical medicine & international health : TM & IH* 2011, **16**(10):1314-1325.

27. Hull HF, Williams PJ, Oldfield F: **Measles mortality and vaccine efficacy in rural West Africa**. *Lancet* 1983, **1**(8331):972-975.

28. Mutua MK, Kimani-Murage E, Ettarh RR: **Childhood vaccination in informal urban settlements in Nairobi, Kenya: who gets vaccinated?** *BMC Public Health* 2011, **11**(1):6.

29. Takum T, Padung D, Joshua V, Manickam P, Murhekar MV: **Programmatic and beneficiary-related factors for low vaccination coverage in Papum Pare district, Arunachal Pradesh, India**. *J Trop Pediatr* 2011, **57**(4):251-257.

30. Hambidge SJ, Newcomer SR, Narwaney KJ, Glanz JM, Daley MF, Xu S, Shoup JA, Rowhani-Rahbar A, Klein NP, Lee GM *et al*: **Timely Versus Delayed Early Childhood Vaccination and Seizures**. *Pediatrics* 2014.

31. Kalan R, Wiysonge CS, Ramafuthole T, Allie K, Ebrahim F, Engel ME: **Mobile phone text messaging for improving the uptake of vaccinations: a systematic review protocol**. *BMJ open* 2014, **4**(8):e005130.

# Tables

Table 1. The Gambian Expanded Programme on Immunization schedule (2011)

| Age | Vaccinations | WHO recommendation* |
| --- | --- | --- |
| Birth | BCG, OPV, Hep B | Birth |
| 2 months | DwPT/Hib/Hep B, OPV, PCV | 6 – 14 weeks |
| 3 months | DwPT/Hib/Hep B, OPV, PCV | 10- 18 weeks |
| 4 months | DwPT/Hib/Hep B, OPV, PCV | 14 – 24 weeks |
| 9 months | Measles, Yellow Fever, OPV | 38 – 52 weeks |
| 18 months | DwPT, OPV | 15 – 24 months |

*http://www.who.int/immunization/policy/immunization_tables/en/

Table 2a. Socio-demographic characteristics of participants

| **Variables** | **n (%)** |
| --- | --- |
| **Gender*** |  |
| Boys | 601 (52.1) |
| Girls | 552 (47.9) |
| **Age group (years)** |  |
| <2 years | 713 (61.8) |
| ≥2 years | 441 (38.2) |
| **Birth place** |  |
| Health Facility | 926 (82.2) |
| Home | 201 (17.8) |
| **Birth Order*** |  |
| ≤2 | 521 (46.4) |
| >2 | 603 (53.6) |
| **Tribe*** |  |
| Mandinka | 406 (35.4) |
| Wolof | 152 (13.2) |
| Fula | 201 (17.5) |
| Jola | 212 (18.5) |
| Others (e.g. Serahule) | 177 (15.4) |
| **Family Type** |  |
| Monogamous | 796 (69.0) |
| Polygamous | 327 (28.4) |
| Single parent | 30 (2.6) |
| **Parents living together*** |  |
| Yes | 925 (80.5) |
| No | 224 (19.5) |

*Missing data

Table 2b: Socio-demographic characteristics of the parents

| **Variables** | **Mother n (%)** | **Father n (%)** |
| --- | --- | --- |
| **Age (years)*** |  |  |
| 15 -20 | 144 (13.0) | 6 (0.9) |
| 21- 30 | 332 (30.0) | 188 (27.1) |
| 31-40 | 387 (35.0) | 325 (46.7) |
| 41- 50 | 243 (22.0) | 176 (25.3) |
|  |  |  |
| **Educational level*** |  |  |
| No formal education | 375 (32.6) | 208 (18.4) |
| Primary school | 453 (39.5) | 386 (34.1) |
| >Primary school | 320 (27.9) | 537 (47.5) |
|  |  |  |
| **Occupation*** |  |  |
| Unemployed | 898 (78.0) | 242 (17.5) |
| Trader | 168 (14.6) | 290 (21.0) |
| Others | 86 (7.4) | 852 (61.5) |

*Missing data

Table 3: Median (IQR) age at vaccination for each vaccine

| Vaccine | Median age at vaccination in days (IQR)* |
| --- | --- |
| BCG | 11 (2, 17) |
| DPT1 | 76 (65, 91) |
| DPT2 | 125 (107, 153) |
| DPT3 | 179 (152, 217) |
| OPV1 | 74 (62, 90) |
| OPV2 | 123 (148, 152) |
| OPV3 | 178 (148, 227) |
| Measles | 293 (279, 318) |

*IQR=Inter Quartile Range

Table 4. Risk factors for delay in receipt of BCG, DPT3, Measles and delay of any vaccines

| **Vaccine/Variables** | **n/N** | **Unadjusted OR**  **(95% CI)** | **Adjusted OR**  **(95% CI)** |
| --- | --- | --- | --- |
| **BCG** |  |  |  |
| **Mother’s Occupation** |  | **p=0.02** | **p=0.02** |
| Unemployed | 58/883 | 1 | 1 |
| Traders | 6/166 | 0.53 (0.23 – 1.26) | 0.46 (0.18 – 1.18) |
| Civil servants | 1/86 | 0.17 (0.02 – 1.22) | 0.18 (0.02 – 1.30) |

| **DPT3** | **n/N** | **Unadjusted OR**  **(95% CI)** | **Adjusted OR**  **(95% CI)** |
| --- | --- | --- | --- |
| **Mother’s Occupation*** |  | **p=0.06** | **p=0.05** |
| Unemployed | 537/894 | 1 | 1 |
| Traders | 112/167 | 1.35 (0.96 – 1.91) | 1.41 (0.98 – 2.02) |
| Civil servants | 45/86 | 0.73 (0.47 – 1.14) | 0.73 (0.47 – 1.16) |
|  |  |  |  |
| **Place of birth*** |  | **p=0.009** | **p=0.03** |
| Health facility | 543/922 | 1 | 1 |
| Home | 138/201 | 1.53 (1.10 – 2.12) | 1.47 (1.05 – 2.07) |
|  |  |  |  |
| **Mode of transportation*** |  | **p=0.001** | **p=0.001** |
| Walking | 302/546 | 1 | 1 |
| Private transport | 4/10 | 0.54 (0.15 – 1.93) | 0.50 (0.14 – 1.83) |
| Public transport | 368/562 | 1.53 (1.20 – 1.95) | 1.54 (1.20 – 1.97) |
|  |  |  |  |
| **Birth Order*** |  | **p=0.007** | **p=0.02** |
| ≤2 | 292/519 | 1 | 1 |
| >2 | 386/601 | 1.40 (1.10 – 1.78) | 1.37 (1.04 – 1.79) |
|  |  |  |  |
| **Delayed DPT1*** |  | p<0.0001 | p=0.52 |
| No | 33/455 | 1 | 1 |
| Yes | 214/693 | 5.7 (3.87 – 8.43) | 0.73 (0.27 – 1.93) |
|  |  |  |  |
| **Delayed DPT2*** |  | p<0.0001 | p<0.0001 |
| No | 60/455 | 1 | 1 |
| Yes | 516/693 | 19.19 (13.92 – 26.45) | 15.14 (10.32 – 22.22) |

| **Measles** | **n/N** | **Unadjusted OR**  **(95% CI)** | **Adjusted OR**  **(95% CI)** |
| --- | --- | --- | --- |
| **Father’s Educational level*** |  | **p=0.005** | **p=0.04** |
| No formal education | 52/203 | 1 | 1 |
| Primary school | 80/379 | 0.78 (0.52 – 1.16) | 0.78 (0.51 – 1.19) |
| >Primary school | 83/531 | 0.54 (0.36 – 0.80) | 0.54(0.35 – 0.82) |
|  |  |  |  |
| **Delayed DPT1*** |  | p=0.0002 | p=0.01 |
| No | 150/890 | 1 | 1 |
| Yes | 68/243 | 1.92 (1.38 – 2.67) | 1.58 (1.10 – 2.26) |
|  |  |  |  |
| **Delayed DPT3*** |  | p<0.0001 | p=0.001 |
| No | 59/450 | 1 | 1 |
| Yes | 158/682 | 2.00 (1.44 – 2.77) | 1.80 (1.27 – 2.56) |

| **Delay in any vaccines** | **n/N** | **Unadjusted OR**  **(95% CI)** | **Adjusted OR**  **(95% CI)** |
| --- | --- | --- | --- |
| **Birth Order*** |  | **p=0.03** | **p=0.05** |
| ≤2 | 313/521 | 1 | 1 |
| >2 | 401/603 | 1.32 (1.03 – 1.68) | 1.27 (0.99-1.64) |
|  |  |  |  |
| **Place of birth*** |  | **p=0.003** | **p=0.005** |
| Health facility | 572/926 | 1 | 1 |
| Home | 146/201 | 1.64 (1.17 – 2.30) | 1.66 (1.16 – 2.37) |
|  |  |  |  |
| **Mode of transportation*** |  | **p=0.002** | **p=0.006** |
| Walking | 325/549 | 1 | 1 |
| Private transport | 3/10 | 0.30 (0.08 – 1.15) | 0.34 (0.06 – 1.88) |
| Public transport | 381/564 | 1.43 (1.12-1.83) | 1.45 (1.12 – 1.86) |

*Missing data
